# Assessing Motivation in Cerebral Palsy During Rehabilitation: A Systematic Review

**DOI:** 10.3390/brainsci16030291

**Published:** 2026-03-05

**Authors:** Daniela De Bartolo, Marco Iosa, Sara Simigliani, Fulvia Di Iulio, Irene Ciancarelli, Giovanni Morone

**Affiliations:** 1 SmArt Lab, IRCCS Santa Lucia Foundation, 00179 Rome, Italy; d.debartolo@hsantalucia.it (D.D.B.); marco.iosa@uniroma1.it (M.I.); 2 Department of Psychology, Faculty of Medicine and Psychology, Sapienza University of Rome, 00185 Rome, Italy; 3 Department of Life, Health and Environmental Sciences, University of L’Aquila, 67100 L’Aquila, Italy; sarasimigliani@gmail.com (S.S.); irene.ciancarelli@univaq.it (I.C.); 4 IRCCS Santa Lucia Foundation, 00179 Roma, Italy; f.diiulio@hsantalucia.it

**Keywords:** motivation, cerebral palsy, neurorehabilitation, standardized measure, participation, engagement, rehabilitation

## Abstract

**Background:** Motivation is widely recognized as a key factor influencing learning and rehabilitation outcomes in children with cerebral palsy (CP). Despite its acknowledged relevance, motivation is rarely assessed systematically in pediatric neurorehabilitation, and there is limited consensus regarding appropriate outcome measures. **Objectives**: This systematic mapping review aimed to examine how motivation-related constructs are assessed in rehabilitation studies involving children with CP, identifying the instruments used and evaluating the extent to which motivation is explicitly measured across different rehabilitation contexts. **Methods**: The review was conducted in accordance with PRISMA guidelines and registered in PROSPERO (CRD420250651843). PubMed and Scopus were searched for studies published between 2013 and 2025. Eligible studies included rehabilitation interventions for children with CP that incorporated a clearly defined motivation-related outcome. Study quality and risk of bias were assessed using Joanna Briggs Institute tools and the RoB 2 tool. **Results**: Nine studies met the inclusion criteria, including 109 subjects, comprising randomized controlled trials and case series. Most studies involved children with mild to moderate motor impairment (GMFCS or MACS levels I–II). Motivation was assessed through heterogeneous approaches, including self-efficacy, mastery motivation, participation, adherence, and intrinsic motivation, with data collected from children, parents, therapists, or dyads. **Conclusions**: Although motivation is frequently cited as a critical component of effective rehabilitation in children with CP, its assessment remains inconsistent and methodologically fragmented. This mapping review, based on a limited and heterogeneous evidence base, highlights the need for standardized, validated, and developmentally appropriate tools to measure motivation-related constructs in pediatric CP rehabilitation.

## 1. Introduction

Cerebral palsy (CP) is the leading cause of cognitive and motor disability in children. CP is defined as a group of permanent, but not unchanging, disorders of movement and posture, which are often accompanied by secondary impairments [1]. Although the neuropathology underlying CP is non-progressive, children with CP may develop a range of secondary associated conditions, including pain, communication difficulty, intellectual disability, vision impairment, dysphagia, fatigue, hip displacement, sleep disorder, decline with aging, and mental health [2].

Recent literature on neuroplasticity suggests that during the development of children with CP, there are consistent changes both at the brain organic and behavioral/functional level and that these changes are largely influenced by personal characteristics, environment, pathology, and therapy undertaken [3,4].

It is recognized that motivation is one of the most significant characteristics influencing rehabilitation outcomes in adults [5]. Motivation had a critical influence also on promoting changes in children’s motor abilities [6] and on developing motor abilities as perceived by the physical therapists [7].

Motivation is a fundamental psychological construct that significantly influences human development, particularly during childhood [8], and it is usually defined as “a person’s willingness to exert physical or mental effort in pursuit of a goal or outcome” [9]. However, motivation is defined in various ways, reflecting the diverse theories that explain this complex process. It is essential to differentiate between intrinsic and extrinsic motivation, as explored in Deci and Ryan’s Self-Determination Theory (SDT) [9]. Intrinsic motivation occurs when a person engages in an activity for personal enjoyment, while extrinsic motivation arises from external rewards and gratification.

According to the conceptual model proposed by Bartlett and Palisano (2002) [7], motivation is a critical determinant of change in motor abilities for children with CP, independent of their health condition. This model emphasizes that children are more likely to engage in activities that are intrinsically motivating, highlighting the importance of aligning rehabilitation goals with the child’s interests to enhance practice [7].

Despite the recognized importance of motivation, there is a lack of systematic assessment of both the child’s and parents’ motivation in daily clinical practice during rehabilitation programs. This study aims to systematically review the rehabilitation literature in individuals with cerebral palsy that includes the assessment of motivation and mapping of “motivation-related constructs”. Specifically, it seeks to identify and critically examine the instruments currently available and used to evaluate motivation within rehabilitation settings for cerebral palsy.

## 2. Materials and Methods

For this Systematic Review, we adopted the Preferred Reporting Items for Systematic Reviews and Meta-Analyses (PRISMA) guidelines [10]. The PRISMA 2020 checklist is provided as Supplementary File S1, detailing the reporting items for this systematic review. The search was restricted to two electronic databases (PubMed and Scopus), to articles published in English, and to a predefined time window (January 2013–February 2025). These limits were applied to ensure feasibility and focus on contemporary rehabilitation approaches; however, they may have led to the omission of relevant studies indexed elsewhere or published in other languages. In addition to database searching, we conducted manual screening of the reference lists of all included articles to identify potentially relevant studies not captured in the initial search. A limited gray literature search was also performed by reviewing conference proceedings and theses indexed within Scopus and by examining reference citations of narrative reviews on pediatric CP rehabilitation. Gray literature sources were included only if they met all predefined eligibility criteria.

The following Medical Subject Headings (MeSH) terms were used: “assessment” OR “evaluation” AND “motivation” AND “cerebral palsy” AND “rehabilitation” OR “motor treatment”.

After removing duplicates manually, the search results underwent a title and abstract screening, applying criteria for inclusion as follows: to be written in the English language, and to include a qualitative or quantitative measure of motivation as a primary or secondary outcome.

Papers with unclear standard measures of motivation were excluded. Standard measure of motivation refers to a clearly described and reproducible assessment method, including validated clinical scales as well as structured quantitative measures (e.g., visual analog scales), provided that the assessment procedure and scoring were explicitly reported. Further entries were added by inspecting the reference list of selected papers included for this review.

Two independent researchers analyzed the quality (SS) and the risk of bias (RoB) (DDB) of the included studies. The selected studies were grouped following the study design, and then a suitable tool for quality appraisal had to be chosen according to the study design. The Cochrane RoB 2 tool was applied to randomized controlled trials [11], evaluating bias across five domains: randomization process, deviations from intended interventions, missing outcome data, measurement of outcomes, and selection of reported results. The Joanna Briggs Institute (JBI) [12] critical appraisal checklist was used for case series, as it provides design-specific criteria suitable for non-comparative studies. The selection of these tools allowed a structured and methodologically coherent assessment across heterogeneous study designs, addressing key domains related to both internal validity and risk of bias [12].

We performed a qualitative synthesis of the included studies because a meta-analysis was deemed inappropriate due to conceptual heterogeneity (different motivational constructs), methodological heterogeneity (RCTs and case series), outcome heterogeneity (self-report, proxy-report, observational, and qualitative), and the absence of common effect size metrics.

The review is registered to PROSPERO with ID CRD420250651843.

## 3. Results

**Figure 1:**
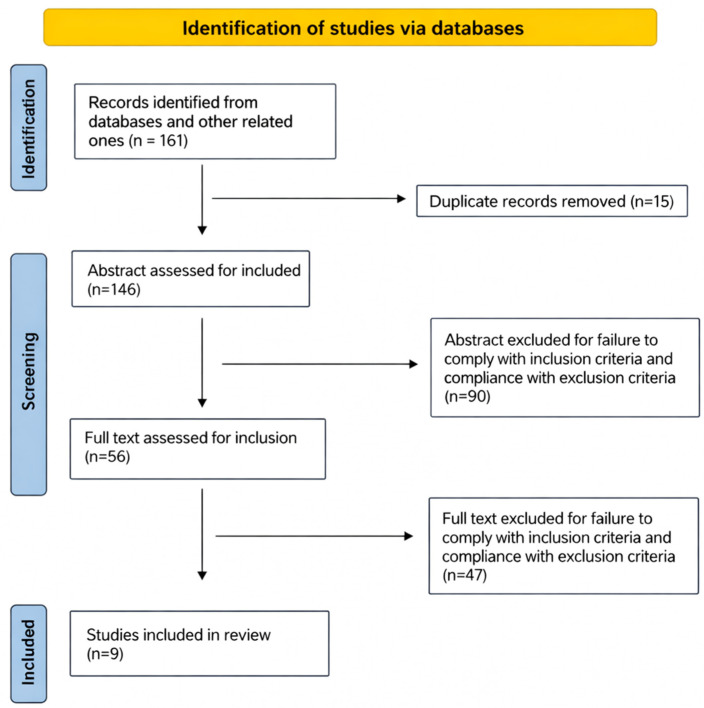
Flowchart of the systematic review.

From 161 results in the above-mentioned database searches, 15 duplicates were removed manually, and 146 results remained for title and abstract screening. A total of 90 records were excluded after abstract reading, while of the remaining 56 papers, 47 were excluded for failure to comply with the inclusion criteria. In addition, both a manual search of reference lists and a manual search of gray literature were conducted. Finally, 9 articles were included in this review, as shown in Figure 1.

The studies included in this review were conducted in Europe (n = 1), North America (n = 3), Oceania (n = 3), and Asia (n = 2). Level of impairment for participants with CP was assessed with the Gross Motor Function Classification System (GMFCS) [13] or the Manual Ability Classification System (MACS) [14] in the case the CP involved only the upper extremities.

Six of the nine studies involved children or adolescents diagnosed with mild CP, while two enrolled participants with an intermediate motor impairment classification, and only one study included participants with all levels of motor impairment. As reported in Table 1, half of the studies [15,16,17,18,19] measured motivation as part of validation projects of innovative devices for the rehabilitation of patients with CP. The remaining studies focused on investigating the effect of designing goal-oriented therapeutic protocols [20] or the efficacy of treatments such as modified constraint-induced movement therapy [21,22] and hippotherapy [23] compared with standard physiotherapy interventions. There is some variability in the duration of treatments, ranging from a minimum of 2 weeks [15] to 26 weeks [21], as well as in the adoption of study design. In fact, 56% of the studies [15,17,20,21,22] adopted an RCT protocol, while the remaining 44% are case series [16,18,19,23]. Of the included studies, Miller et al. 2016 [21] and 2015 [22] refer to the same study whose data are analyzed at two different time points (13 and 26 months of treatment).

### 3.1. Motivation-Related Constructs Measures

**Figure 2:**
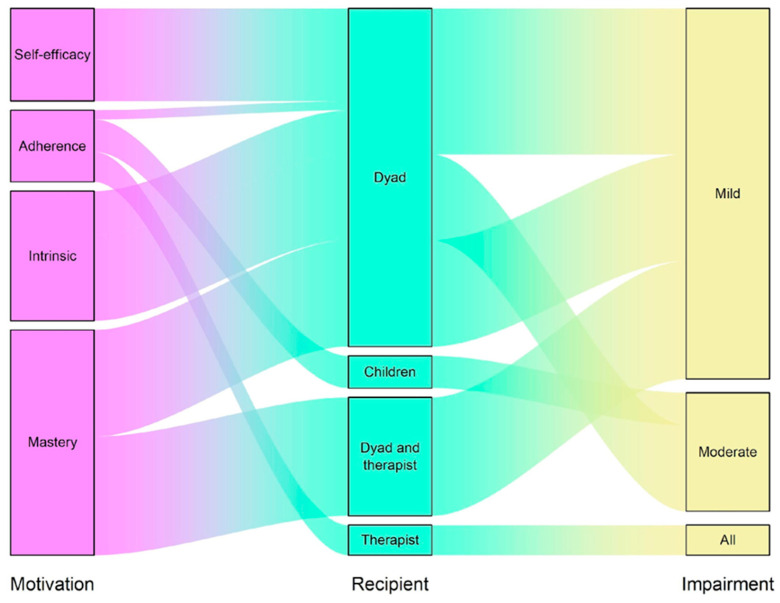
The alluvial plot shows how motivation-related constructs were assessed across studies involving children with CP. The diagram links the type of motivation targeted by the researcher (left), the recipient who completed the motivation assessment tool (center), and the level of motor impairment of the participating children (right). The width of each connection reflects the number of participants included in each study. Dyad refers to both caregivers and the children involved.

According to the aim of this systematic review, we selected studies in which the assessment of motivation-related constructs of children involved in a rehabilitation protocol was performed. Figure 2 shows an alluvial plot of how motivation-related constructs were assessed by the studies. Across the included studies, a variety of instruments were used to investigate motivation toward motor therapy in children with cerebral palsy, with substantial heterogeneity in both constructs and measurement approaches. The most consistently used structured instrument was the Dimensions of Mastery Questionnaire (DMQ) [24], employed to assess mastery motivation and task persistence. The DMQ was primarily administered as a caregiver proxy report [21,22], although one study also included adolescent self-report alongside caregiver ratings [23]. One study [18] assessed motivational experience within activity contexts using the Self-Reported Experiences of Activity Settings (SEAS) [25], a child-report questionnaire measuring psychological engagement, perceived choice and control, and enjoyment during activity participation, thereby capturing motivational aspects of therapy-related settings.

Motivation-related behaviors during therapy were also evaluated using observational measures, including the Pediatric Volitional Questionnaire (PVQ) [26], which captures volitional and exploratory behaviors during therapy tasks [22], and the Pittsburgh Rehabilitation Participation Scale (PRPS) [27,28], used by therapists to rate engagement, effort, and motivation during rehabilitation sessions [19]. One study [15] involving 40 participants assessed motivation as self-efficacy in the accomplishment of the therapy goals set using the Canadian Occupational Performance Measure (COPM) [28] administered both to parents and children, scoring also performance and satisfaction for each therapy session. Several studies adopted combined or indirect approaches, embedding motivation-related constructs within broader outcome frameworks. For example, Reedman et al. (2019) [20] assessed motivational self-beliefs using the Belief in Goal Self-Competence Scale (BiGSS) [29] alongside a modified Canadian Occupational Performance Measure, with ratings provided collaboratively by children and caregivers when appropriate. In addition to structured tools, non-standardized measures were frequently reported, including Likert-scale enjoyment ratings [19], visual analog scales (VAS) [15], and open-ended [16] or semi-structured interview questions [17] exploring enjoyment, engagement, or perceived motivational factors related to therapy participation.

### 3.2. Quality Assessment and Risk of Bias Analysis

**Figure 3:**
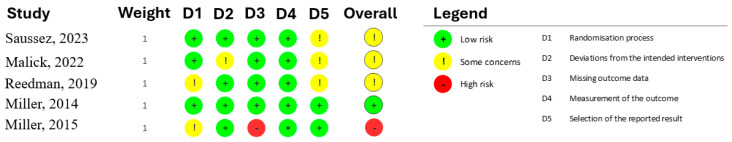
Risk of bias analysis (RoB 2 Tool) of RCT studies [15,17,20,22,30].

The RoB-2 assessment reported in Figure 3 indicates that most RCT domains were rated as low risk of bias; however, caution is warranted in interpreting these ratings. Several studies relied on subjective, proxy-reported, or therapist-rated outcomes, and blinding of outcome assessors was often limited or unclear, particularly for motivation-related constructs. Other concerns emerged mainly in the selection of reported results, where one study displayed a high risk of bias. Therefore, although formal RoB2 ratings were generally favorable, the potential influence of measurement subjectivity and limited blinding should be considered when interpreting these findings.

Among the included RCT studies, the role of motivation still emerges variably but remains central as either an implicit driver of engagement or, more rarely, as an explicit therapeutic target. The recent RCT by Malick et al. (2022) [17] evaluated three different augmented-reality (AR) games (Balance It, Bubble Pop, and Scoop’d) in 30 children (6–12 years) with spastic hemiplegic CP over 8 weeks. All groups showed significant improvements in upper-extremity function (DASH) and balance (Pediatric Balance Scale), with the “Balance It” game yielding somewhat stronger balance gains. This suggests that AR-based playful, movement-centered interventions can successfully engage children in repeated practice, potentially enhancing motivation through fun, real-time feedback, and self-directed movement. In the context of participation-focused therapy, the ParticiPAte CP trial of Reedman et al. [20] used a self-determination theory approach (motivational interviewing, child-selected leisure goals), improving goal performance and participation even if MVPA changes were modest, indicating that autonomy-supportive design fosters engagement and behavior change. Meanwhile, the Saussez et al. (2023) [15] study about HABIT-ILE and REAtouch^®^ showed that integrating a semi-immersive virtual device into an intensive upper-limb training program yields comparable functional outcomes to traditional therapy, with motivation inferred from good adherence rather than formally measured. And finally, the Miller et al. (2016, 2015) [21,22] mastery motivation study demonstrated through the Dimensions of Mastery Questionnaire that children’s baseline mastery motivation predicts upper-limb therapy outcomes, underscoring the importance of intrinsic motivational traits for treatment success, beyond mere motor capacity. Altogether, these RCTs reinforce that AR/VR and participation-oriented therapies can support high-dosage or high-engagement rehabilitation in CP but also highlight the ongoing need for consistent, validated measures of motivation to compare effectiveness across studies.

The methodological quality of the non-RCT studies is reported in Table 2; it is assessed using the JBI Critical Appraisal Checklist for Case Series [12] and was generally low, with all four studies demonstrating a high risk of bias.

Overall, these studies contribute important preliminary insights into motivational processes during technology-based or context-specific interventions, but their low methodological rigor means that conclusions must be interpreted cautiously and considered hypothesis-generating rather than confirmatory. Chan-Víquez et al. (2023) [16] illustrate that home-based videogame training (Bootle Blast) can elevate intrinsic motivation through fun, challenge, and autonomy, with children frequently exceeding self-directed playtime goals, though motivation was characterized qualitatively rather than measured quantitatively. MacIntosh et al. (2020) [18] integrate experiential data through the SEAS and show that a 4-week biofeedback videogame program is both engaging and feasible for youth with CP, highlighting how enjoyment and personal involvement interact with functional change. Reubens and Silkwood-Sherer (2016) [23] uniquely track motivation using the DMQ-17 within a hippotherapy and home-PT program, documenting increases in mastery motivation alongside gains in functional endurance and mobility. Hung et al. (2018) [19] further support the motivational appeal of motion-controlled gaming (Kinect2Scratch), reporting high adherence and early increases in engagement relative to traditional therapy, while also noting waning motivation for some children after novelty decreases. Together, these studies underscore the motivational potential of playful, technology-mediated, or personally meaningful therapeutic contexts while also demonstrating the value of standardized motivation instruments still inconsistently applied across studies.

### 3.3. Strengths and Limitations of the Identified Motivation Measures

A critical appraisal of the instruments identified reveals that the DMQ appears to be the most theoretically grounded tool, as it directly targets mastery motivation and intrinsic persistence. However, its predominant reliance on caregiver proxy report introduces potential reporting bias and may not fully capture the child’s subjective motivational experience. The psychometric properties of DMQ have been assessed in different studies and translated into different languages. Its validity was tested in a wide age range, going from 6 months up to 19 years old, with a moderate-good inter-rater reliability and moderate test–retest reliability [30]. The COPM, although widely used and clinically meaningful, primarily assesses perceived goal performance and satisfaction, which represent behavioral outcomes or consequences of motivation rather than intrinsic motivational processes themselves. The validity of the COPM was reported in many different age ranges, including children between 1 and 7.5 years old, with good inter-rater reliability and moderate test–retest reproducibility [31]. Other observational tools, such as the PVQ and the PRPS, offer valuable insight into engagement during therapy sessions, yet they remain dependent on clinician interpretation and are therefore inherently subjective. The PVQ has been mainly validated in the age range between 3 and 7 years. It has a moderate to high inter-rater reliability, but with less information about test–retest properties [30]. Moreover, few of these instruments have been specifically validated for children with more severe motor impairment or significant cognitive limitations, raising concerns about their applicability across the full spectrum of cerebral palsy severity.

## 4. Discussion

The aim of this study is to examine how motivation-related constructs are currently assessed within rehabilitation research involving children with CP. Specifically, we sought to determine whether and how motivation is measured in neurorehabilitation settings to describe the instruments used for this purpose, mapping of “motivation-related constructs”.

We found that two questionnaires were mainly used to assess motivation in children with CP. One is the Dimensions of Mastery Questionnaire [32]. The DMQ assesses mastery motivation by having a parent or teacher rate their perceptions of the child’s behavior in mastery contexts. Mastery motivation is a multifaceted, intrinsic psychological force that stimulates an individual to attempt to master a skill or task that is at least moderately challenging for him or her. Another instrument used was the Canadian Occupational Performance Measure, COPM [28], which was mainly defined to measure two scores, one related to the performance and one to the satisfaction with performance, in the field of occupational therapy. Despite not psychometrically validating on children, it has investigated the opinions of children and their parents about the use of COPM in pediatric groups [32]. Considering this finding, we might distinguish between motivation, defined as the “intrinsic force” or psychological drive (measured by the DMQ), and participation/performance, which represents the behavioral outcome or manifestation of that drive (measured by the COPM) [32]. One study [19] used the Pittsburgh Participation Rehabilitation Scale [33], which is based on a single item compiled by the therapist. This scale was validated on a sample with an age range between 20 and 96 years, so it was not previously validated on pediatric populations. Considering this finding, it is essential to differentiate motivation as an internal psychological construct, engagement as its observable behavioral expression during task performance, and participation as the broader functional outcome reflecting involvement in meaningful activities.

In early childhood, motor skill development is not a purely motor process but relies heavily on cognitive resources such as attention, working memory, and executive functions, even during seemingly simple activities like walking [34]. How this cognitive process is involved might depend on motivation, which, for this reason, is a key component of the subjects’ learning process and motor behavior changes [35].

The engagement is an important observable behavioral expression for the execution of rehabilitation, for its performance, and in terms of both temporal and attentional engagement [36]. This assumption is particularly important during the rehabilitation of neurodevelopmental disorders, as it is very often based on neurodevelopmental treatment (NDT) with goal-oriented exercises that require frequent feedback towards the child to enhance motivation and increase compliance with motor training [36]. For this reason, in recent years, several new techniques, mostly based on technological tools, were introduced to increase motivation during children’s rehabilitation: serious game, non-immersive virtual reality, and augmented reality are examples [37,38].

However, despite the acknowledged importance of motivation and the growing number of interventions designed to enhance it, our review highlights that motivation is still rarely assessed in a systematic and comprehensive manner. Motivation outcomes are frequently underreported in pediatric rehabilitation trials, and when they are included, there is considerable heterogeneity in the instruments and methods used.

Similar gaps have been reported in rehabilitation research involving adults with central neurological conditions [5], as well as in broader pediatric rehabilitation populations [39], suggesting that this issue is not specific to CP but reflects a more general limitation in rehabilitation research.

Consistent with previous similar research, Ref. [38] most of the studies included in this review included motivation as an outcome measure for the validation of a new therapy (i.e., virtual reality, serious game, and CIMT) designed to provide joy and fun as an add-on to the conventional exercises. While incorporating amusement into therapy is undoubtedly beneficial for sustaining motivation in children, enjoyment alone should not substitute for a structured assessment of motivational processes. Without appropriate measurement tools, it remains difficult to determine how motivation contributes to treatment effects or to control for its influence when comparing different interventions.

Unlike previous reviews [40], which included heterogeneous pediatric populations with different neurodevelopmental and neurological conditions, our study deliberately focused exclusively on children with cerebral palsy. This methodological choice was intended to reduce population-related heterogeneity and to address a key limitation of earlier work, in which the inclusion of multiple disabilities may have obscured condition-specific aspects of motivation and its assessment.

By restricting the analysis to children with CP, we aimed to improve the interpretability of findings in relation to the distinctive cognitive–motor profile of this population. Most of the included studies involved children with relatively high functional levels, predominantly classified as GMFCS or MACS levels I or II, with only two exceptions [18,20]. This is consistent with the assumption that the effective use of motivation as a lever for learning and motor improvement requires at least partially preserved cognitive and motor abilities, allowing children to actively engage in goal-directed and feedback-based rehabilitation tasks [41].

Nevertheless, despite our efforts in securing a more homogeneous population, the present review faces important limitations. Considerable variability persists across study designs, types of motivational assessment tools, age ranges of participants, and rehabilitation approaches investigated. This residual heterogeneity reflects the current state of the literature and underscores the absence of shared standards for assessing motivation in pediatric CP rehabilitation. Rather than representing a methodological weakness alone, this variability highlights the complexity of the theoretical construct of motivation and reinforces the need for more unified and theoretically grounded assessment frameworks in future research.

Our results showed that often the target of the measure is the child, therapist, or parents, or the dyad, leading to a heterogeneity of information but reflecting different aspects of the child’s motivation, useful especially for different levels of functioning. From a clinical perspective, a future standardized assessment tool should likely integrate multiple viewpoints, capturing the child’s subjective experience alongside observations from parents and therapists, each of whom can provide unique and complementary insights. Motivation outcomes, although important, are frequently subjective and often secondary endpoints, increasing the likelihood of measurement bias. In addition, we have to account for the limited generalizability of findings only to less affected individuals. This implies that clinicians and researchers need motivation measures applicable across the full spectrum of CP severity (GMFCS/MACS III–V).

This review has methodological limitations. The search strategy was restricted to two databases (PubMed and Scopus), English-language publications, and a defined time frame (2013–2025). Although these criteria were selected to ensure feasibility and focus on contemporary rehabilitation literature, relevant studies indexed in other databases or published in other languages may have been missed. Another limitation is the absence of a single theoretical framework guiding the assessment of motivation. Although this choice increased heterogeneity among the identified tools, it was necessary to comprehensively map how motivation is currently conceptualized and measured in pediatric CP rehabilitation. This exploratory approach allowed us to capture the breadth of existing practices rather than restricting the analysis to a predefined theoretical model. Finally, it is important to note that risk of bias and internal validity, although related, are not interchangeable concepts. While the RoB 2 tool focuses primarily on bias within randomized designs, the JBI checklist evaluates broader methodological rigor in non-comparative studies.

Overall, our findings support the need for a more systematic and rigorous assessment of motivation in pediatric rehabilitation. There is growing evidence that higher motivation is associated with better rehabilitation outcomes [42], yet the mechanisms underlying this relationship remain insufficiently understood. However, these findings should be interpreted cautiously, given the small number of included studies, their methodological heterogeneity, and the limited rigor of several case series designs. Future research should prioritize the development of standardized, validated, and clinically feasible tools for measuring motivation in children with CP. In parallel, further studies are needed to clarify how motivation interacts with neuroplasticity, learning processes, and functional severity, and how these relationships vary across different levels of impairment.

## 5. Conclusions

Available evidence suggests that motivation-related constructs likely play a relevant role in rehabilitation learning processes in children with cerebral palsy; however, their assessment remains limited, heterogeneous, and inconsistently operationalized. Future research should prioritize the development and validation of theory-driven, psychometrically robust tools specifically designed to assess motivation-related constructs in children with CP. An ideal instrument should distinguish intrinsic motivational processes from behavioral outcomes, integrate multiple informants (child, caregiver, and clinician), be applicable across CP severity, demonstrate robust psychometric properties, and be sensitive to change over the course of rehabilitation. Ensuring clinical feasibility will be essential to promote its routine implementation in pediatric neurorehabilitation settings.

## Figures and Tables

**Table 1:** The table shows demographic information obtained by the included studies. In the column of sample size and age, we reported the number of participants enrolled with the range of minimum-to-maximum age expressed in years. Clinical assessment refers to classification ranging from Level I (mild impairment) to IV (severe impairment). VR stands for virtual reality, UE and LE are for upper and lower extremities, respectively; mCIMT is for modified constrained induced movement therapy. Refs. [21,22] are for two time points of the same study published in two different papers. COPM is for the Canadian Occupational Performance Measure; SEAS is for the self-reported experiences of activity settings; PPRS stands for Pittsburgh Rehabilitation Participation Scale, and BiGSS is for the Belief in Goal Self-Competence Scale.

First Author (Year)	Country	Study Design	Sample: Size and Age Range (Years)	Participant Levels (GMFCS/MACS)	Intervention Type and Side	Duration of Treatment	Outcome Measure
Saussez et al. (2023) [15]	Belgium	RCT	40 (5–18)	I–II	VR technology (UE)	2 weeks	COPM, unstructured open questions
Chan-Víquez et al. (2023) [16]	Canada	Case series	4 (7–17)	I–II	Exergame (UE)	14 weeks	Semi-structured interview
Malick et al. (2022) [17]	Pakistan	RCT	30 (6–12)	I–II	Augmented Reality (LE)	8 weeks	Unstructured open questions
MacIntosh et al. (2020) [18]	Canada	Case series	19 (8–18)	I–II	VR technology (UE)	4 weeks	SEAS
Hung et al. (2018) [19]	Taiwan	Case series	13 (5–15)	I–V	Exergame (UE)	12 weeks	PPRS
Reedman et al. (2019) [20]	New Zealand	RCT	37 (8–12)	I–III	Goal oriented physiotherapy (UE)	16 weeks	BiGSS, COPM
Miller et al. (2016) [21]	Australia	RCT	44 (5–16)	I–II	mCIMT (UE)	26 weeks	DMQ, PVQ
Miller et al. (2015) [22]	Australia	RCT	46 (5–16)	I–II	mCIMT (UE)	13 weeks	DMQ
Reubens et al. (2016) [23]	USA	Case series	1 (13)	I–II	Hippotherapy	10 weeks	DMQ

**Table 2:** Qualitative assessment using the Joanna Briggs Institute (JBI) Critical Appraisal Checklist for Case Series.

Study	JBI Items Met (Out of 10)	Overall Risk of Bias	Main Strengths	Main Limitations
Chan-Víquez et al., 2023 [16]	3/10	High	Clear description of intervention; rich qualitative insight into motivational processes.	No clear inclusion criteria; non-consecutive sampling; small sample; outcomes not standardized; short duration; descriptive analysis only.
MacIntosh et al., 2020 [18]	4/10	High	Use of validated measures (SEAS, COPM, and AHA); detailed reporting of participant characteristics; structured single-case design.	Single participant; absence of comparator; short follow-up; limited external validity; no inferential statistics.
Reubens & Silkwood-Sherer, 2016 [23]	4/10	High	Clear clinical description; use of standardized motivation measure (DMQ-17); detailed intervention reporting.	Case report (N = 1); no inclusion criteria or series structure; limited follow-up; inability to separate intervention effects from natural variation.
Hung et al., 2018 [19]	3/10	High	Good feasibility data; detailed reporting of engagement and participation during training.	Lack of standardized motivation measures; no consecutive or complete inclusion; short intervention; descriptive analysis; early motivational “novelty effect” not controlled.

## Data Availability

No new data were created or analyzed in this study.

## Supplementary Materials

The following supporting information can be downloaded at: https://www.mdpi.com/article/10.3390/brainsci16030291/s1. File S1: PRISMA checklist.
